# Effect of standardized patient simulation-based pedagogics embedded with lecture in enhancing mental status evaluation cognition among nursing students in Tanzania: A longitudinal quasi-experimental study

**DOI:** 10.1186/s12909-024-05562-4

**Published:** 2024-05-26

**Authors:** Violeth E. Singano, Walter C. Millanzi, Fabiola Moshi

**Affiliations:** Department of Nursing Management and Education, The University of Dodoma, Dodoma, Tanzania

**Keywords:** Cognition, Standardized patient, Simulation pedagogics

## Abstract

**Background:**

Nurses around the world are expected to demonstrate competence in performing mental status evaluation. However, there is a gap between what is taught in class and what is practiced for patients with mental illness among nursing students during MSE performance. It is believed that proper pedagogics may enhance this competence. A longitudinal controlled quasi-experimental study design was used to evaluate the effect of using standardized patient simulation-based pedagogics embedded with a lecture in enhancing mental status evaluation cognition among nursing students in Tanzania.

**Methods:**

A longitudinal controlled quasi-experimental study design with pre-and post-test design studied 311 nursing students in the Tanga and Dodoma regions. The Standardized Patient Simulation-Based Pedagogy (SPSP) package was administered to the intervention group. Both groups underwent baseline and post-test assessments using a Interviewer-adminstered structured questionnaire as the primary data collection tool, which was benchmarked from previous studies. The effectiveness of the intervention was assessed using both descriptive and inferential statistics, specifically the Difference in Difference linear mixed model, and the t-test was carried out using IBM Statistical Package for Social Science (SPSS) software, version 25.

**Results:**

The participant’s mean age was 21 years ± 2.69 with 68.81% of the students being female. Following the training Students in the intervention group demonstrated a significant increase in MSE cognition post-test, with an overall mean score of (*M ± SD* = 22.15 ± 4.42;p = < 0.0001), against (*M ± SD* = 16.52 ± 6.30) for the control group.

**Conclusion:**

A significant difference exists in the levels of cognition, among nursing students exposed to Mental Status Evaluation (MSE) materials through Standardized Patient Simulation-Based Pedagogy (SPSP) embeded with lectures. When MSE materials are delivered through SPSP along with lectures, the results are significantly superior to using lectures pedagogy alone.

## Introduction

The most prominent and dominant strategy used to diagnose a mental health problem in a clinical setting is Mental status evaluation MSE [1]. The type of diagnosis is based on the chief signs and symptoms, and treatment is agreed upon accordingly. The MSE is data received using information gathered by the psychiatrist, clinician, and nurse from direct inquiries and passive assessment during the interview to determine the patient’s actual mental state. The purpose of evaluating the range of mental functions and behaviors at a particular moment gives crucial information for diagnosis and determining the disease’s severity, trajectory, and responsiveness to treatment. Countries such as the U.S. practice mental status evaluation as a diagnostic tool for the diagnosis of mental illness, and the rest of the world uses similar cataloging from the American Psychiatric Association [2]. Nursing students are expected to demonstrate competence in performing mental status evaluation. However, there is a gap between what is taught in class and what is practiced for patients with mental illness among nursing students during MSE performance. Classroom and clinical pedagogies, such as lecture role play and demonstrations, are implemented to facilitate MSE competencies among nursing students [3]. Scholars have reported conventional pedagogics such as lectures, demonstrations, and portfolios to be dominantly used in facilitating MSE learning among nursing students [4]. The predominant use of conventional pedagogy has been linked to anxiety, frustration, stress, and fear in nursing students when they encounter mentally ill patients during their clinical rotation [5, 6].

Educators and health workers argue that these abilities are inadequate to provide evidence-based mental health nursing care. They may thus lead to prolonged hospital stays, remissions, drug-resistant and long-term adverse drug effects in mentally ill patients [7]. The study was conducted on the practices of the nursing students throughout clinical teaching in mental health hospitals and stated that there is a mismatch between theory and practice, insufficient instruction approaches, and an absence of person-mode nurses and coaching staff to facilitate MSE learning for nursing students appropriately [8]. A study by [9] on designing instruction to teach MSE reported that nursing students who taught MSE using conventional clinical pedagogics demonstrated inabilities to diagnose patient conditions plan patient care, prevent injury to patients and others, and provide specific management. Moreover, findings from [10] on the discrepancy between what occurs internally and externally in student mental health nursing showed a significant mismatch between theoretical mental health content knowledge and practical skills when nursing students are developed using conventional clinical pedagogy.

International and national organizations respond to Sustainable Development Goal number four, target number four (SDG), by emphasizing training institutions and teaching hospitals to adopt and implement innovative pedagogics in facilitating MSE learning for learners [11]. The incorporation of standardized patient simulation-based pedagogy (SPSP) as suggested by other scholars [12, 13], appears to demonstrate academic potential, such as enhancing learners’ cognitive and empowering them with self-efficacy when performing MSE. Simulation offers a chance to make cases more challenging without endangering clients, families, or students, as nursing students in clinical practice are frequently tasked with working with amicable and amenable clients and families [14]. The SP comes to life in front of the learners in the state-of-the-art lab. Students can practice their diagnosis and develop therapeutic clinical expertise in the laboratories, which are offered in a friendly environment [15].

Similarly, a pilot study using a mixed method was done in Baccalaureate nursing education in the US to examine the use of SPSP compared with the traditional hours used for learning mental health, showing nursing students who received SPSP showed increased confidence and cognition about mental health by 25% compared to traditional hours [16]. Good MSE cognition among nursing students may ultimately lead to timely and appropriate diagnosis and, thus, positive mental health outcomes for mental illness patients. While the adoption and implementation of SPSP are popular in other countries, published scholarly works are scarce about it in clinical nursing education for MSE cognition among nursing students in Tanzania. It may be time to invest in research about the effect of SPSP embedded with lecture on enhancing MSE cognition among nursing students in this country.

## Method and materials

The methodology of this study complied with national and international research ethics. Moreover, the study was conducted by the University of Dodoma’s institutional postgraduate guidelines and standards.

The purpose of the current research was to evaluate the impact of standardized patient simulation-based pedagogy (SPSP) linked with lectures on mental status evaluation cognition among nursing students in Tanzania. To accomplish this, a longitudinal quasi-experimental study design was implemented.

### Study population

The target demographic was made up of students enrolled in diploma nursing programs in the regions of Tanga and Dodoma. The study involved 311, diploma nursing students (the age between 16 and 32 years). The reason for selecting middle college nursing students is that they constitute a big population of the future nursing force, which is expected to deliver nursing mental health services in the peripheral community. This study believed that skills provided to the nursing students were beneficial for them since it is targeted to be delivered to the large population for timely diagnosis of mental illness disorders, which most of the population are living in remote areas with inadequate mental health services.

### Sampling procedure and technique

The purposive sampling technique was used to sample nursing schools from two regions, 5 nursing schools from Dodoma Central zone and 2 nursing schools from Tanga in the Northern zone, where 311 nursing students sampled and (109) were in the intervention group and (202) nursing students were in the control group then the proportional calculation was done to get the required number of participant’s in each nursing school whereby the simple random sampling were done to select the requires number of participants in each class. After being explained the purpose, and benefit of this study, nursing students who were willing to participate in this study and signed the written informed consent form were included in this study.

### Proportional for the intervention group

1 $${\rm{n = }}\left( {{\rm{n/N}}} \right){\rm{ Nh}}$$

Whereby n = Total number of sample sizes for each group whether interventional Control croup.

N = Total number of students in both classes.

Nh = Total number of students in each class.

Two nursing schools from Tanga were Tanga College of Health and Allied Sciences (TACOHAS) with a total number of students of 97, college A, and Korogwe Nursing Training Center (KNTC) with a total number of students of 71, college B.

The proportion for college A;

A+B = 168

N = 109

N = 168

Nh = 97 and 71.

nA = (109/168) 97 = 63.

Therefore, the number of participants in College A was 63.

nB = (109/168) 71 = 46.

Therefore, the number of participants in College B was 46 (making a total sample size of 109 for the intervention group).

### The proportion for the control group in the Dodoma region

Five colleges offering Diplomas in nursing from Dodoma are DECCA College of Health and Allied Sciences (DECCA COHAS) with a total number of nursing students 60; Dodoma Institute of Health and Allied Sciences (DIHAS) with a total number of nursing students 81; Saint John’s University with a total number of nursing students of 45; Mvumi Institute of Health and Allied Sciences (MIHAS) with a total number of students of 19; Kondoa School of Nursing with a total number of 47;

Therefore,

Total number of students = 252.

nC = (217/252) 60 = 52.

nD = (217/252) 81 = 70.

nE = (217/252) 45 = 39.

nF = (217/252) 19 = 16.

nG = (217/252) 47 = 40.

Which makes a total sample size of 217.

**Fig. 1:**
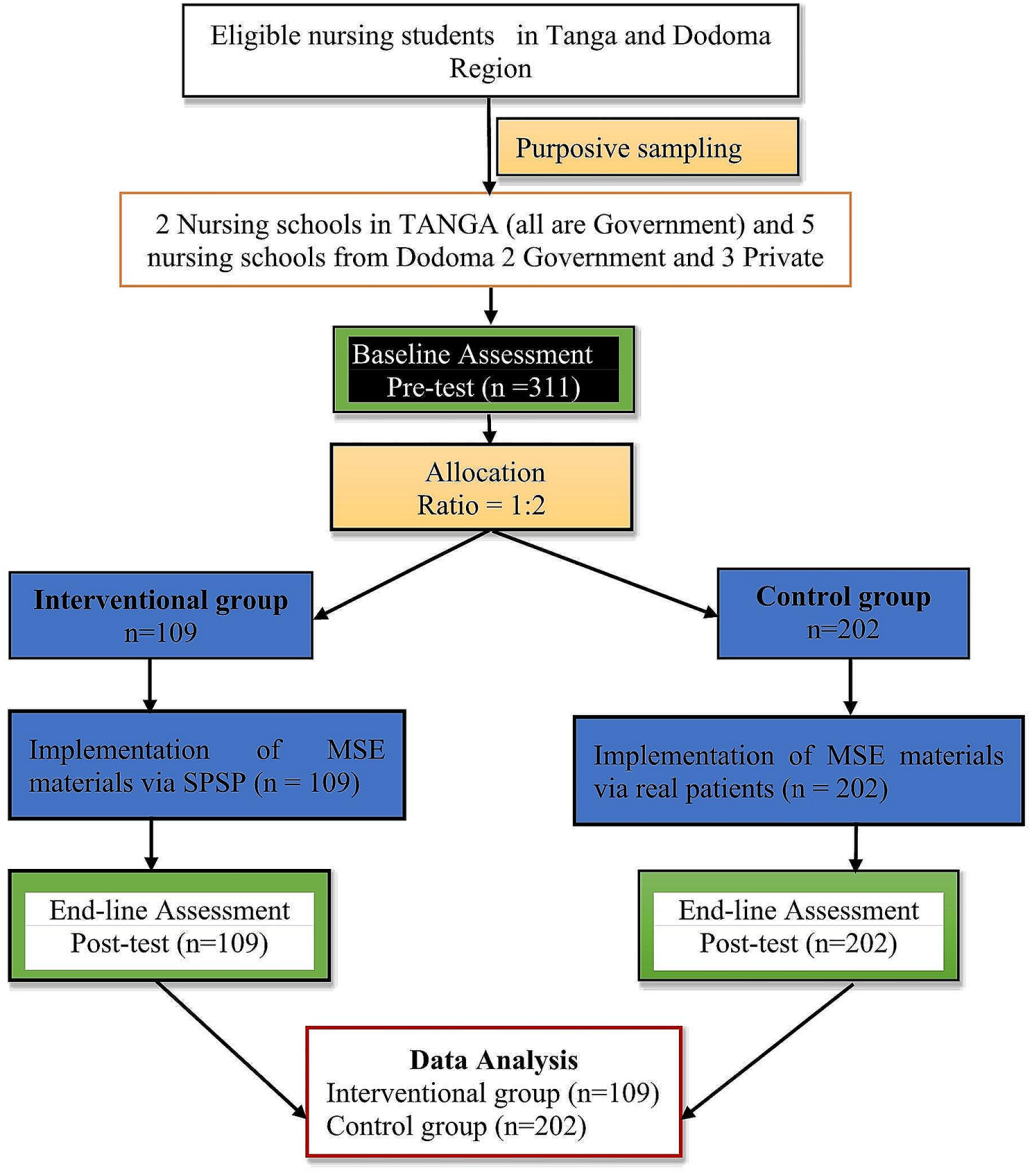
Study design flow diagram Source: Study plan (2022)

The required number of participants was obtained through proportional calculation. To select participants, a simple random sampling method was employed by listing the names of students on pieces of paper. The selection was made by choosing participants for every 10th number on the list until the required number was reached, Fig. 1 illustrates this. To prevent contamination, interventional and control groups were assigned to different regions, and participants were not informed about the other study sites. Additionally, the researcher’s assistant was kept unaware of whether participants were part of the control or intervention groups.

### Sample size estimation

**Fig. 2:**
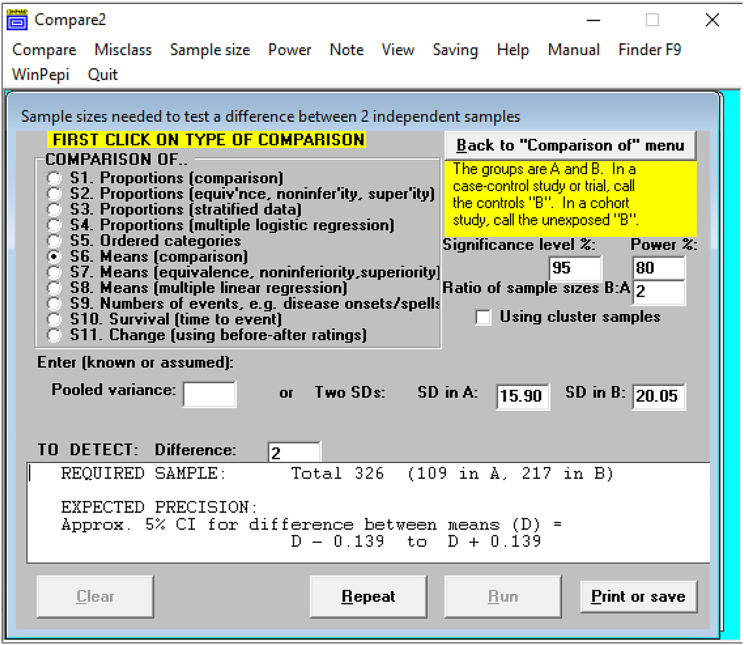
WinPepi program for sample size calculation. Source: Study plan (2022)

The sample size n for this study was determined using WinPepi software version 11.65 [17]. Findings from the study on simulation-based learning in psychiatry for undergraduates at the University of Zimbabwe Medical School [18] showed a pre-session mean score of 15.90 and a post-session mean score of 20.05. With a sample size of the effect size of 2, a significance of 95% confidence interval of 5% significance level, and a power of the study of 80%, the ratio of the sample size B: A is a ratio of 1:2.As shown in Fig. 2, therefore, sample size (n) = 326 Participants (109 in A and 217 in B). This program has been used by different scholars and reported to have statistical validity and reliability in studies [19–21].

### Data collection procedure

After obtaining the necessary permissions, an available classroom was designated for the study. The Principal Investigator then introduced the study’s objectives to the participants. Once informed consent was obtained, the students were seated in separate chairs to prevent any potential copying or sharing of responses. Data collection was carried out by interviewer-administered structured questionnaire, with the trained researcher’s trainer. The Principal Investigator was present to provide clarifications when necessary. Once completed, the questionnaires were collected by the trained researcher trainer and securely stored in a locked cupboard by the Principal Investigator.

### Data collection tool

This study employed a standardized structured questionnaire benchmarked from previous studies [22] with 33 items modified from a literature review. The adopted questionnaire for cognition has a test re-test approach which is used to assess the dependability of the study instrument (alpha reliability = 0.770, test-re-test reliability = 0.880). Therefore, the questionnaires used for data collection in this study consisted of two parts: Part “A” collected demographic characteristics profiles of the study participants, Part “B” assessed participants’ MSE cognition (28 items),

### Validity

Nursing professionals were given the first draft of the instrument, and they were asked to reply to the open-ended questions, propose any changes they believed should be made, and suggest any additional items they thought should be added. Items having a relevancy score of less than 0.7 were removed, and adjustments to the wording were made to the expert’s suggestions. For face and construct validity, a preliminary draft was examined by a second nursing expert from a nursing faculty. Students in their second year of nursing (*n* = 33) provided comments on the tool’s usefulness. After comments from the experts and the nursing students, there were 5 questions from cognition questions that lacked face validity or content validity were removed. A total of 28 questions on cognition remain.

### Reliability

To verify the tool’s capabilities for producing the expected results, a pilot study of 10% of the sample size was conducted. The statistical program for the Social Solution (SPSS) software version 25 was used to scale the results from the pilot study. The overall Cronbach’s alpha of cognition was 0.736. As recommended by previous scholars, a Cronbach’s Alpha (α) of ≥ 0.7 was considered a significantly reliable tool for the actual field data collection.

### Variable measurement

A structured questionnaire benchmarked from previous studies was used to measure the variable pre- and post-intervention to test cognition. Cognition of MSE was measured using multiple-choice open-ended questions for baseline assessment and immediate 1-week post-intervention, the test had 28 questions for assessing MSE cognition with three domains including (2-questions) on the concept of MSE, (4-questions) on the content of MSE and (22-questions) on MSE implementation. Scores per each correct response ranged from ֞ 0 ֞ point for a wrong response to.

֞ 1 ֞ point for a correct response, and the highest score of MSE cognition were computed as a sum of each item, then cognition was explained as ֞ adequate cognition ֞ for participants who scored 50% and above, and the lowest score was explained as ֞ inadequate cognition ֞ for participants who scored < 50%. The domain of MSE cognition was also measured separately based on the total score that the nursing students scored out of the total score assigned to each domain then, the mean difference between the two groups was measured using a paired t-test.

### The SPSP intervention

Table 1 shows the prescription of the intervention training. The intervention took 4 weeks to facilitate both MSE theory and practice. Topics of the MSE materials included a definition of MSE and steps in performing MSE to identify a client with mental illness. Two sessions were conducted in a week, lasting 120 min each. They were facilitated during the morning hours and were negotiated with the principles of the respective colleges. Two sessions were implemented to cover the MSE theoretical and practical sessions, respectively. Both English and Swahili were used alternatively at the convenience of research trainers and participants. The intervention group learned MSE using an SPSP embedded with a lecture compared to the control group, which learned the same MSE materials using lectures and real patient pedagogies. The rationale behind choosing these two approaches was to assess the impact of the intervention on two groups : those who were exposed to the MSE materials via SPSP embedded with lecture and went on to the skills laboratory to interview the SP who is trained and coached to portray sign and symptoms of mental illness, and those who were exposed through MSE lecture methods and actual patients in general medical wards without symptoms of mental illness. Upon completion of the data analysis, a comparison was made between the subjects who were exposed to the MSE materials through SP and the subjects who were exposed to the actual patient who did not exhibit any symptoms of mental illness. Before the intervention, participants in both groups were matched in their sociodemographic profiles, such as age, sex, education level, entry qualification, and marital status, to ensure their similarities before intervention. Pre-tests were then administered to participants to establish their baseline MSE cognition.

The MSE intervention focused on the area where nursing students struggled with technique questions to assess and determine if the patient exhibited the characteristics of hallucination, illusion, delusion, derealization, depersonalization, and insight, terms that can used commonly. To help nursing students understand that what the patient demonstrated or explained reflected the question asked, that failing to probe precisely what the patient was experiencing may lead to the wrong MSE conclusion, and that the SP was trained to answer the questions asked to reflect the reality of what the patient was suffering from, how these questions were asked was given more consideration.

**Table 1:** Prescription of SPSP training in an intervention group

	Lecture	SPSP materials
**Contents**	MSE theory	pictures, scenarios, problem-based questions,
**Venue**	Classroom	Classroom and Skills Laboratory
**Facilitator**	Trained Research trainers	Trainer Research trainers
**Mode of delivery**	Face- to -face delivery	Face- to face- delivery
**Teaching method**	Through lecture	Gallery work, each one teaches one, group discussion, pictorial method, and simulation
**Dose**	1 session for theory	2 session1(60–120) theory 1 practical
**Materials**	Projector, computer, notes	Projector, computer, pictures, scenario, flip chart and marker pen, notebook, training package, lesson plan, checklist
**Frequency**	Only 1 session per week	3 sessions per week 1session for the classroom and 2 sessions for the simulation
**Time for each session**	60–120 min depending on the amount of content or materials	120 min for the classroom, 15 min for interviewing the SP, and 10 min for debriefing
**Sitting plan**	Two by two sitting arrangement	Semi-cycle sitting arrangement
**In class activities**	Listen and taking some notes	Group discussion, each teach one, Gallery work
**Mode of assessment**	No session evaluation	After the simulation, the students will be required to volunteer to tell others what they have learned but also at the end of the training package there will be a question on evaluating the training

*Source* Study plan 2022

### Recruitment and training of SP and research trainers

#### Training of SP

Professional actors who know mental health, work at a mental health facility, or have a family relative who has a mental illness, or encountered a person with a mental health problem and who were willing to help the student learn and be able to retain the script of the scenario was recruited as SP. Principles for preparing SPSP were found in the association of Standardized patient education standards, and practice [23] was applied to ensure SPSP is a safe work environment and training for role portray and feedback to students during debriefing. The agreed-upon formula, primary goals, duties, materials, and structure of the mental health scenario were covered during a weekly 2-hour training class. This class included instruction on scenario reading, guidance in verbal interaction techniques, input on the scenario, debriefing strategies, and discussions on how to reduce learner anxiety during the simulation experiences.

Before the rehearsal, each SP was provided with a scenario that outlined the signs and symptoms of a mentally ill patient. This scenario encompassed the various domains of MSE, with specific questions and answers to which the SP was required to respond in each domain. Emphasis was placed on the domains that nursing students commonly encountered difficulties with during mental status evaluation and clinical practice. For instance, they were trained on how to assess mood and affect, illusions and hallucinations, depersonalization and derealization, orientation, memory, intelligence, insight, and judgment. However, not all SPs were required to portray all domains of symptoms. This is because it’s uncommon for one patient to exhibit all possible symptoms simultaneously. Additionally, having all the symptoms portrayed by the SPs might lead to an exaggeration of the true symptoms of a real patient.

The SPs were thoroughly rehearsed using scenario scripts, and the research team, mental health experts, and nurse tutors who specialize in teaching mental health subjects reviewed their performances. The portrayal of the client’s character was observed, and the experts addressed any areas that required clarification or correction. Out of the four SPs who were willing to participate in this study, two were able to effectively portray the signs and symptoms of a mentally ill patient and were selected for the actual fieldwork implementation.

### Implementation of MSE materials in an SPSP

Nursing students were assigned to the interventional group (typical education plus SPSP), which first completed both pre-tests before getting intervention. The MSE lecture method was taught to the students on the first day of the training by the researcher trainers focused on the definition of MSE, steps on performing MSE, and how to perform MSE to identify patient with mental illness disordes. The nursing students were then introduced to the simulation on the following day, and they were informed that the simulation would take place in a skills laboratory, nursing students were invited to the prepared skills laboratory, Students were seated on the semi-cycle sitting plan for easy visualization of the simulation, and then SP together with the nursing student who acted as a nurse were seated at the center and the researcher trainer was there to provide any assistance needed by students during simulation. Interventional students participated in two-hour simulation sessions, with a break in between to prevent student fatigue. Each group consisted of 5 to 8 students. Following the simulation pre-briefing on the scenario was done by the researcher trainer to make sure that they understood the whole simulation process, and SPSP orientation was included in each simulation. Thereafter each nursing student was provided with a checklist of the MSE categories to make a follow-up to what had been assessed during the simulation. SP was brought to the skills laboratory by his relatives dressed in dirty loose- jogging tracksuits and his hair was messy with a history of abnormal behavior characterized by abusive language, over-talkative, threatening his mother and others, reduced sleep during the night, grandiose delusion, persecutory delusion and hearing unknown voices, one nursing student was chosen from the class for each simulation to play the nurse role on how to perform MSE to the patient with abnormal behavior by using the technique and procedures learned during the lecture methods, and the other was designated as an observer. The positions of nurse and observer were available to all students. The duration of each simulation was 15 min, followed by a 10-minutes structural debriefing.

### Evaluation of MSE materials in an SPSP

The three-part debrief paradigm, which entails defusing, identifying, and developing [24], served as the framework for the debriefing sessions. The trained researcher trainer was offered SPSP one-on-one organized time for debriefing immediately following each simulation exercise to examine psychological problems in role acting and how students’ emotional states influence their conduct and communication. To encourage cooperative learning, SP and nursing student observers discussed what they had noticed about communication and evaluation methods. Students playing nurses’ roles were encouraged to speak about their experiences. SP provided feedback via formative and summative methods that involved face-to-face engagement. The trained researcher trainer commented on the student’s responses.

### Data analysis

The IBM statistical package of Social Science (SPSS) computer software program version 25 was used to analyze data. The frequency distribution table was used for data cleaning to ensure that all data was recorded accurately. To go through the data, labels had to be applied, value had to be checked and re-assigned for the open-ended questions, noise had to be checked, and the erroneous spellings verification for nominal response had to be rewritten. Additionally, the baseline and end-line data were combined and added during the procedure of the calculation of the important outcome A descriptive and inferential analysis was conducted based on the study’s goal. To calculate the frequencies and the percentage of each participant’s distribution between the two groups, a descriptive analysis was performed to examine participant characteristics Bar chats, mean values, and averages as well as tabular data, were all included in the descriptive evaluation. The pre-post mean score, and post-test mean score, for both the interventional and control groups were compared using the independent samples t-test. To evaluate the effect of the SPSP embedded with a lecture on MSE cognition, among nursing students from baseline to end line, the inferential analysis involved the differences in difference (DID) analysis using a Linear mixed model. A 95% Confidence interval set at a 5% (≤ 0.05) significance level was used to reject the null hypothesis. Results from the parameter multiple measurements were taken into consideration by models, and the groups were considered as fixed influences.

### Difference–in–difference (DID) analysis for inferential analysis

By eliminating the confounding variables, difference-in-difference (D-I-D) analysis enables the comparison of changes over time in the results between interventions. The DID design examines the difference between the treatment groups by measuring the change in results between two-time intervals (pre and post) for the intervention and control groups, then subtracting one from another. In this research, the impact of the intervention on cognition change score was evaluated using difference-in-difference analysis using a linear mixed method. The outcomes of the variables’ repeated measurements were taken into account by the model. Interventions were regarded as having fixed effects in this analysis. The following formula is used to present the general fixed-effect DID mixed model

2 $$\eqalign{& {Y_{it}}\; = \;{\beta _0}\; + \;{\beta _1}\;*\;Time\; + \;{\beta _2}\;*\;Treatment\; + \; \cr & {\beta _3}\;*\;Time\;*\;Treatment\; + \;{\varepsilon _{it}} \cr & \Rightarrow \;{\mu _t}\; = \;E({Y_{it}})\; = \;{\beta _0}\; + \;{\beta _1}\;*\;Time\; + \;{\beta _2}\; \cr & *\;Treatment\; + \;{\beta _3}\;*\;Time\;*\;Treatment \cr}$$

Time is an empty variable for the period, denoted as 1 when the outcome analysis was completed in the final stage and 0 for benchmark evaluation. Here, ***Y***_***it***_ is the final result for participant *i* at time *t*. This variable acts as a substitute variable for the intervention group. The combined parameter Time* Treatment is the relationship between time and the intervention, this **ε**_**it**_ is also the amount of error for the participant *i* outcome measurements at the time *t.* The value of the intercept in the equation given parameter β_0_, represents the mean outcome value for the group receiving the intervention at the baseline measurement. β_1_ is the change in an intervention group’s mean outcome variable between the baseline and the end line Parameter β_2_ represents the variation in the mean result variable across individual interventions. The estimate and inference of the difference-in-difference between the two groups are provided by the coefficient of the interaction between groups.

## Results

### Social demographic characteristics among nursing students

Distribution of the similarity of demographic characteristics among nursing students between intervention and control groups at the baseline. Table 2 reported that among the participants (*n* = 311), who indicated their age 34.86% (*n* = 38) for intervention and 54.46% (*n* = 110) for control were ranged between 21 and 32 years old with their age distribution (*p* = 0.0510) between groups, for those who indicated their gender 57.80% (*n* = 63) were female in intervention group and 68.81% (= 139) were from control group with (*p* = 0.0521) of their gender distribution between groups. However, for those who are single were many in both groups compared to those who are married 95.41% (*n* = 104) for intervention and 97.45% (*n* = 191) with (*p* = 0.3373*) of their distribution between marital status. The distribution of form four education entrance was higher compared to others with 76.15%(*n* = 83) for control and 59.90% (*n* = 121) for intervention with (*p* = 0.0540) of their education level distribution between groups. Among all participants, 99.07% (*n* = 107) in intervention and 84.69% (*n* = 166) in control showed interest in nursing with (*p* = 0.0601) of their interest distribution between groups.

**Table 2:** Comparison of Baseline Characteristics between intervention and control group among nursing students (*n* = 311)

Variable	Intervention *n* (%)	Control *n* (%)	Chi-Square	*P*-Value
Age (M ± SD = 21years ± 2.83)	21.04 ± 2.69	21.52 ± 2.96		
Minimum	16.00	16.00		
Maximum	32.00	32.00		
**Age category in years**			3.8967	0.0510
≤ 20	71(65.14)	92(45.54)		
>20	38(34.86)	110(54.46)		
**Sex**			3.7725	0.0521
Male	46(42.20)	63(31.19)		
Female	63(57.80)	139(68.81)		
**Physical/psychological disability**				0.1013*
Yes	1(0.93)	9(4.69)		
No	107(99.07)	183(95.31)		
**Marital status**				0.3373*
Married	5(4.59)	5(2.55)		
Single	104(95.41)	191(97.45)		
**Entry education**			4.2795	0.0540
Form four	83(76.15)	121(59.90)		
Form six/college	26(23.85)	81(40.10)		
**Interest in nursing professional**				0.0601*
Yes	107(99.07)	166(84.69)		
No	1(0.93)	30(15.31)		

*Source* study findings (2023)

### The effect of standardized patient simulation-based pedagogics embedded with lecture on MSE cognition among nursing students in Tanzania

As shown in Table 3. below, the cognition pretest score of the concepts of MSE in the intervention group was (*M ± SD* = 0.87 ± 0.84) and the control group was *M ± SD* = 0.81 ± 0.79, *p* = 0.5341, the post-test results were *M ± SD* = 1.33 ± 0.73 for the intervention group, and the control was *M ± SD* = 1.17 ± 0.76; *p* = 0.0785, so there is a marked change of MSE content from both groups. However, the MSE content on the baseline was (*M ± SD* = 2.63 ± 1.06) for the intervention and (*M ± SD* = 2.42 ± 0.95; *p* = 0.0700) for the control group, the end line score for the intervention group was (*M ± SD* = 3.39 ± 0.80) and control group was (*M ± SD* = 2.85 ± 0.98; *P* < 0.0001), baseline findings for implementation of MSE Intervention group scored (*M ± SD* = 11.4 ± 3.00) and control group scored (*M ± SD* = 9.85 ± 3.96; *p* = 0.0076 for the pre-test intervention group scored (*M ± SD* = 17.42 ± 3.90) control group scored (*M ± SD* = 13.29 ± 4.58; p = < 0.0001).

The overall pretest was (M = 13.05, SD = 4.63) for intervention group, (*M ± SD* = 12.11 ± 5.21), *p* = 0.1189 from control group posttest cognition was (*M ± SD* = 22.15 ± 4.42) for intervention and (*M ± SD* = 16.52 ± 6.30; p = < 0.0001) for control group. There is a significant change in cognition for the intervention and control group for the post-test. According to the substantial mean changes between the pre-test and post-test scores in all categories (Concepts, Content, Implementation, and Overall cognition) for the intervention group, it appears that the intervention has had a significant change effect on the cognition of nursing students in general. A small amount of progress is also seen in the control group, but overall, the intervention group exhibits more development.

**Table 3:** Overall mean score changes and difference in MSE cognition between baseline and end line among Nursing students (*n* = 311)

Variable	Mean ± Std	Mean diff	t-Value	*P*-Value
**Concepts pre test**		0.06 ± 0.81	0.62	0.5341
Intervention	0.87 ± 0.84			
Control	0.81 ± 0.79			
**Concepts post-test**		0.16 ± 0.75	1.77	0.0785
Intervention	1.33 ± 0.73			
Control	1.17 ± 0.76			
**Content pre test**		0.21 ± 0.99	1.80	0.0700
Intervention	2.63 ± 1.06			
Control	2.42 ± 0.95			
**Content post-test**		0.55 ± 0.92	4.95	< 0.0001
Intervention	3.39 ± 0.80			
Control	2.85 ± 0.98			
**Implement pre test**		1.18 ± 3.65	2.69	0.0076
Intervention	11.04 ± 3.00			
Control	9.85 ± 3.96			
**Implement post-test**		4.12 ± 4.35	7.90	< 0.0001
Intervention	17.42 ± 3.90			
Control	13.29 ± 4.58			
**Overall cognition pre-test**		0.93 ± 5.02	1.56	0.1189
Intervention	13.05 ± 4.63			
Control	12.11 ± 5.21			
**Overall cognition post-test**		5.63 ± 5.71	8.28	< 0.0001
Intervention	22.15 ± 4.42			
Control	16.52 ± 6.30			

*Source* Study findings (2023)

### Findings of nursing student’s cognition mean score between baseline and end line (*n* = 311)

**Fig. 3:**
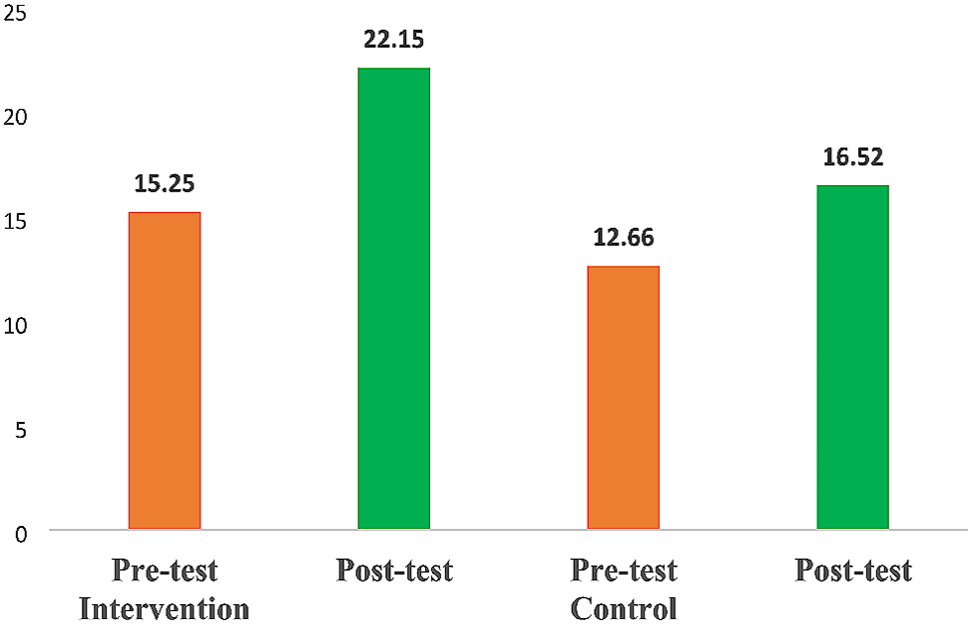
Findings of nursing student’s cognition mean score between baseline and end-line. Source: Field data (2023)

The finding shows that the mean cognition increased from base to end line between the interventional group and the control group. As shown in Fig. 3 mean score of nursing student cognition increased by (*M ± SD* = 22.15 ± 4.42) for the intervention group, whereas cognition in the control group increased by (*M ± SD* = 16.52 ± 6.30). This implies that the change in cognition mean score from baseline to end line was higher in the intervention group than in the control group.

### DID analysis for MSE cognition among nursing students in Tanzania

The fitted model results are presented in Table 4. The findings indicate that there was a significant improvement in cognition from the baseline to the end line, as indicated by a p-value of < 0.0001. The coefficient for the Difference-in-Differences (D-I-D) analysis, comparing the intervention group to the control arm, was 4.6950. This suggests that the change in cognition from baseline to end line was significantly higher in the intervention group compared to the control group.

**Table 4:** Parameter estimates of the linear mixed model for the difference in differences analysis of MSE cognition among nursing students in Tanzania

Effect	Estimate	Standard Error	Pr > |t|
Intercept	12.1139	0.3529	< 0.0001
**Time**			
End line	4.4059	0.4930	< 0.0001
Baseline	Reference		
**Treatment**			
Intervention	0.9320	0.5961	0.1189
Control	Reference		
Time*Treatment			
	**D-I-D coefficients**		
Effect	Estimate	Standard Error	P-Value
Intervention Vs. Control	4.6950	0.8327	< 0.0001

*Source* Study findings (2023)

## Discussion

The study’s results establish a strong correlation between the impact of Standardized Patient Simulation-Based Pedagogy embedded with lecture (SPSP) and the cognition scores of nursing students. In the final analysis, nursing students exposed to standardized patient-based simulation materials displayed a significantly higher level of cognition regarding Mental Status Evaluation (MSE) when compared to the control group. This outcome aligns with a study conducted at university of Queens Canada on the impact of SPSP in psychiatric nursing on mental health education, which demonstrated a significant cognition improvement [25].Additionally, nursing students who interacted with standardized patients (SP) during interviews were able to relate what they had learned from the designed teaching pedagogy during the simulation, the simulation’s method of delivery provided nursing students with ample time to interview the SP. The study done by [26] on the use of SPS to train new nurses supporting this findings that new nursing students cognition improved higher compared to control group whose not exposed to the SPS.

During simulation process in case where clarification was required or certain behaviors were not well understood by the learners, students could request the SP to repeat the behavior, but also the skills of the trained researcher on the delivering of the content contributed to the increasing nursing students’ cognition. This aligns with the findings of a study conducted in Australia to explore the effect of SPSP on mental health education. The study reported that students who used SPSP for teaching scored higher, felt safer, and experienced reduced anxiety levels during examinations, as demonstrated in research [27, 28]. Given the challenge of exposing nursing students directly to realistic patients without prior practice in a skills laboratory, exposing nursing students in SPS demonstrated a significant higher level of cognition this changes are due to the fact that students were able to control the learning environment during the simulation, a similar study was conducted in Baccalaureate nursing education in the US to examine the use of SPSP compared to traditional hours dedicated to learning mental health. The study found that student nurses who received SPSP demonstrated a 25% increase in confidence and cognition about mental health compared to traditional instructional hours, as highlighted in the research by [16]. Observing others successfully perform Mental Status Evaluation (MSE) using Standardized Patients (SP) and receiving encouraging feedback from colleagues and facilitators played a pivotal role in boosting nursing students’ cognition. This research aligns with study done to compare SPS versus mannequins in mental health simulation, which posits that cognition is influenced by positive simulation modalities, guidance through observational learning, approval, and inspiration [29]. The training program encouraged students to focus on acquiring the necessary knowledge, and the briefing provided during simulation on how MSE should be conducted contributed to building students’ MSE cognition.

However, This outcome aligns with a study conducted by [30] on the use of SPSP in psychiatric nursing, which demonstrated a substantial improvement in nursing students’ understanding compared to traditional teaching methods. Specifically, the study reported an 80% increase in cognition acquisition when utilizing SPSP as opposed to conventional approaches. These findings are consistent with a study conducted by [31], which implemented various active teaching methods during simulation to enhance nursing students’ knowledge. Additionally, the manner in which SPs were trained to accurately portray signs and symptoms of patients was instrumental in this process. Furthermore, the design of the SP teaching materials fostered collaboration among nursing students, encouraging each student to actively participate in classroom activities. This collaborative approach played a vital role in enhancing their MSE cognition. These findings are consistent with the work of several scholars, such as [32] and [33], who have emphasized the significant contribution of peer-to-peer education in boosting nursing students’ sense of cognition.

## Conclusion

The findings of this study suggested that using SPSP embedded with lectures will help increase nursing student MSE cognition among nursing students in Tanzania. This is because there is no skills laboratory for nursing students to practice before encountering a real patient, and the practicum sites for nursing students to practice mental health services, especially MSE, are few. For this reason, nursing students are required to travel far from their institution to practice. This is contrary to the Tanzania curriculum, which states that nursing students should practice in the skills laboratory before going to the clinical. Standardized patients in teaching mental status evaluation is a useful pedagogical method and increases the cognition of the nursing students, while it’s difficult to use real patients because it may cause inconveniences to the patient and the learner. MSE is challenging to assess because it cannot be directly assessed as a physical disease. Nursing students require the technique of performing MSE to get the real symptom from the patient.

### Strength of the study

To improve the performance of nursing students, the study addressed clinical pedagogical deficiencies in clinical mental health nursing education on Mental status evaluation to better manage and diagnose people with mental diseases promptly. However, the study has managed to use a control group and enough sample to increase the validity of results and power of the study on the effect of SPSP and their outcome.

### Suggestion for further studies

Future researchers should include this training among nursing students at higher institutions. Future studies should address the problems with the study’s design and expand on some of the topics that were not fully explored in this one. Based on the study’s shortcomings, there were several implications that another study might take into account.

## Limitations of the study

The generalization of the study findings among nursing students in Tanzania will be difficult since the calculated sample size was 326 and the participants who were willing to participate in this study during actual data collection was 311, even though the response rate was 95%. The results of the study cannot be used to determine whether they apply to all Tanzanian nursing students this is because study participants were the nursing students from the middle college who are pursuing diplomas in nursing from Dodoma and Tanga Regions, and excluded the university students who are also learning MSE and are expected to deliver MSE service within the community, and they also suffer from a lack of stimulation of MSE in a skill-based environment. Consequently, results must be examined and analyzed carefully while considering them. The study employed purposive sampling that cannot tell exactly that the selected participants present the sample of nursing students in Tanzania. However, the Study did not show the separate effect of lecture as embedded in the SPSP training materials and how much contributed to the outcome of interest.

## Data Availability

The datasets that are used or analyzed in the current study are available from the corresponding author on reasonable request via wcleo87@gmail.com or walter.millanzi@udom.ac.tz.

## Abbreviations

MSE: Mental Status Evaluation
SP: Standardized patient
SPSS: Statistical Package of Social Sciences
SPSP: Standardized patient Simulation Pedagogics
UDOM: University of Dodoma
U.S.: United states
WHO: World Health Organization
